# A Central Role for TRPM4 in Ca^2+^-Signal Amplification and Vasoconstriction

**DOI:** 10.3390/ijms23031465

**Published:** 2022-01-27

**Authors:** Tamás Csípő, Ágnes Czikora, Gábor Á. Fülöp, Hajnalka Gulyás, Ibolya Rutkai, Enikő Pásztorné Tóth, Róbert Pórszász, Andrea Szalai, Kata Bölcskei, Zsuzsanna Helyes, Erika Pintér, Zoltán Papp, Zoltán Ungvári, Attila Tóth

**Affiliations:** 1 Division of Clinical Physiology, Department of Cardiology, Faculty of Medicine, University of Debrecen, 4032 Debrecen, Hungary; tamas.csipo@gmail.com (T.C.); agnes.czikoraa@gmail.com (Á.C.); fulop.gabor.aron@gmail.com (G.Á.F.); hajnal.gulyas11@gmail.com (H.G.); irutkai@tulane.edu (I.R.); pasztore@med.unideb.hu (E.P.T.); pappz@med.unideb.hu (Z.P.); 2 Doctoral School of Kálmán Laki, University of Debrecen, 4032 Debrecen, Hungary; 3 Department of Pharmacology and Pharmacotherapy, Faculty of Medicine, University of Debrecen, 4032 Debrecen, Hungary; robert.porszasz@gmail.com (R.P.); szalai.andrea@med.unideb.hu (A.S.); 4 Doctoral School of Pharmaceutical Sciences, University of Debrecen, 4032 Debrecen, Hungary; 5 Department of Pharmacology and Pharmacotherapy, Medical School, University of Pécs, 7624 Pécs, Hungary; bolcskeikata@outlook.com (K.B.); zsuzsanna.helyes@aok.pte.hu (Z.H.); erika.pinter@aok.pte.hu (E.P.); 6 Szentágothai Research Centre, University of Pécs, 7624 Pécs, Hungary; 7 HAS-UD Vascular Biology and Myocardial Pathophysiology Research Group, Hungarian Academy of Sciences, 4032 Debrecen, Hungary; 8 Vascular Cognitive Impairment and Neurodegeneration Program, Oklahoma Center for Geroscience, Department of Biochemistry and Molecular Biology, University of Oklahoma Health Sciences Center, Oklahoma City, OK 73104, USA; zoltan-ungvari@ouhsc.edu; 9 International Training Program in Geroscience, Department of Public Health, Semmelweis University, 1089 Budapest, Hungary; 10 The Peggy and Charles Stephenson Cancer Center, University of Oklahoma Health Sciences Center, Oklahoma City, OK 73104, USA; 11 Department of Health Promotion Sciences, College of Public Health, University of Oklahoma Health Sciences Center, Oklahoma City, OK 73104, USA

**Keywords:** transient receptor potential, transient receptor potential melastatin-4, blood pressure regulation, vascular smooth muscle, Ca^2+^ signaling

## Abstract

Transient receptor potential melastatin-4 (TRPM4) is activated by an increase in intracellular Ca^2+^ concentration and is expressed on smooth muscle cells (SMCs). It is implicated in the myogenic constriction of cerebral arteries. We hypothesized that TRPM4 has a general role in intracellular Ca^2+^ signal amplification in a wide range of blood vessels. TRPM4 function was tested with the TRPM4 antagonist 9-phenanthrol and the TRPM4 activator A23187 on the cardiovascular responses of the rat, in vivo and in isolated basilar, mesenteric, and skeletal muscle arteries. TRPM4 inhibition by 9-phenanthrol resulted in hypotension and a decreased heart rate in the rat. TRPM4 inhibition completely antagonized myogenic tone development and norepinephrine-evoked vasoconstriction, and depolarization (high extracellular KCl concentration) evoked vasoconstriction in a wide range of peripheral arteries. Vasorelaxation caused by TRPM4 inhibition was accompanied by a significant decrease in intracellular Ca^2+^ concentration, suggesting an inhibition of Ca^2+^ signal amplification. Immunohistochemistry confirmed TRPM4 expression in the smooth muscle cells of the peripheral arteries. Finally, TRPM4 activation by the Ca^2+^ ionophore A23187 was competitively inhibited by 9-phenanthrol. In summary, TRPM4 was identified as an essential Ca^2+^-amplifying channel in peripheral arteries, contributing to both myogenic tone and agonist responses. These results suggest an important role for TRPM4 in the circulation. The modulation of TRPM4 activity may be a therapeutic target for hypertension. Furthermore, the Ca^2+^ ionophore A23187 was identified as the first high-affinity (nanomolar) direct activator of TRPM4, acting on the 9-phenanthrol binding site.

## 1. Introduction

Members of the transient receptor potential melastatin (TRPM) channel subfamily are expressed on vascular smooth muscle cells, and are involved in vascular smooth muscle function [1]. One member, the TRPM4 is a nonselective cation channel [2] that is known to play a critical role in the development of cerebrovascular myogenic tone [3,4,5]. Myogenic tone of the cerebral arteries is essential for the autoregulation of intracranial blood flow, and in vivo suppression of TRPM4 leads to the impairment of this autoregulation in a rat model [6]. TRPM4 is activated by the increase in the intracellular Ca^2+^ concentration and is impermeable to Ca^2+^ [7]. TRPM4 elicits rapid desensitization to intracellular Ca^2+^, which is inhibited by phosphatidylinositol-4,5-bisphosphate [8,9]. Protein kinase C (PKC) modulates its Ca^2+^ sensitivity and activity through phosphorylation in vascular smooth muscle cells [3,10].

TRPM4 is present in a wide variety of tissues including T-lymphocytes [11], pancreatic β-cells [12], and cardiomyocytes [13], in addition to vascular smooth muscle cells [4]. TRPM4 has two major roles, depending on the expression of L-type Ca^2+^ channels (LTCCs). In nonexcitable cells (which do not express LTCCs, e.g., T-lymphocytes) TRPM4 activation creates a membrane depolarization that limits further Ca^2+^ entry by decreasing the driving force of Ca^2+^ currents [14]. In excitable cells that express LTCCs (e.g., vascular smooth muscle cells, cardiomyocytes) TRPM4 evokes an increased influx of Ca^2+^ ions through the LTCCs mediated by membrane depolarization [4,14]. While the presence and function of TRPM4 in cerebral arteries is widely accepted [4,6,15], its role in other arteries is debated [16,17].

TRPM4 has several pharmacological inhibitors, but no specific TRPM4 agonist with sufficient affinity has been reported. One of the most specific inhibitors of TRPM4 is 9-phenanthrol (9-hydroxi-phenanthrene), which is widely used in TRPM4-targeted research [18,19,20].

The hypothesis of this study was that TRPM4 makes an essential contribution to smooth muscle contraction by amplifying intracellular Ca^2+^ elevations. The role of TRPM4 was studied via the Ca^2+^ ionophore A23187 (reported as a nonspecific agonist) and 9-phenanthrol (antagonist) in various isolated vascular beds and in the systemic circulation of the rat.

## 2. Results

### 2.1. Pharmacological Inhibition of TRPM4 Results in a Dose-Dependent Decrease in Blood Pressure

**Figure 1:**
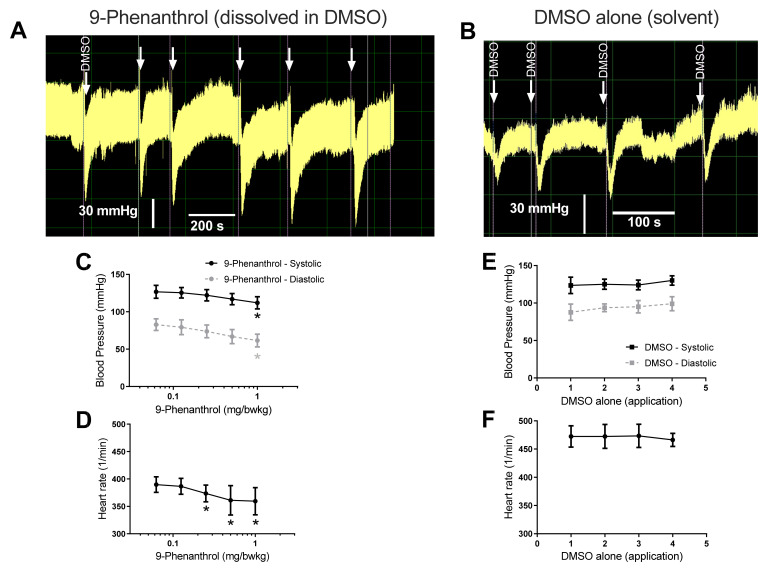
Intravenous administration of TRPM4 inhibitor 9-phenanthrol decreased arterial blood pressure and heart rate. Invasive blood pressure measurement of rats was performed via cannulation of the left carotid artery. TRPM4 inhibitor 9-phenanthrol was administered (shown by unlabeled arrows, in Panel **A**) through a venous catheter placed in the jugular vein. Consecutive (cumulative) applications were initiated when the blood pressure response stabilized (reached a steady state), usually after 2–3 min. The peak effects observed during the bolus applications are plotted on the graphs. (**A**) Representative recording; (**B**) representative recording upon the application of the solvent DMSO alone; (**C**) systolic and diastolic blood pressure values at the peak of the transient effect of 9-phenanthrol; (**D**) heart rate at the peak of the transient effect of 9-phenanthrol, where the systolic pressure values were determined. Statistically significant effects upon the cumulative application of 9-phenanthrol (compared to the effect of DMSO (solvent) alone (labeled arrow in panel **A**), determined by Friedman test) are labeled by the asterisks; (**E**) effects of repeated DMSO (solvent) application on blood pressure; and (**F**) heart rate of rats. Graphs show the mean ± SEM. Six animals were tested with 9-phenanthrol (*n* = 6, panels **A**,**C**,**D**) and a separate six animals were tested with DMSO alone (*n* = 6, panels **B**,**E**,**F**).

The effect of TRPM4 inhibition was investigated by i. v. administration of 9-phenanthrol into the jugular veins of rats. 9-Phenanthrol caused a dose-dependent, transient decrease in blood pressure (Figure 1A). Both systolic (from 131 ± 8 to 112 ± 8 mmHg, *n* = 5, *p* < 0.01, Figure 1C) and diastolic (from 81 ± 9 to 62 ± 9 mmHg, *p* < 0.01, Figure 1C) blood pressure decreased (as compared to the blood pressure after vehicle –DMSO-administration, prior to the application of 9-phenanthrol, indicated by the arrows in Figure 1A). A significant decline in heart rate (from 402 ± 14 to 359 ± 25, *n* = 5, *p* < 0.05, Figure 1D) was also observed in parallel with the blood pressure changes. Note that repeated applications of solvent (DMSO alone) did not result in declines in blood pressure or heart rate (Figure 1B,E,F).

### 2.2. TRPM4 Inhibition Abolishes Myogenic Response to Pressure Elevation in Skeletal Muscle Arteries

**Figure 2:**
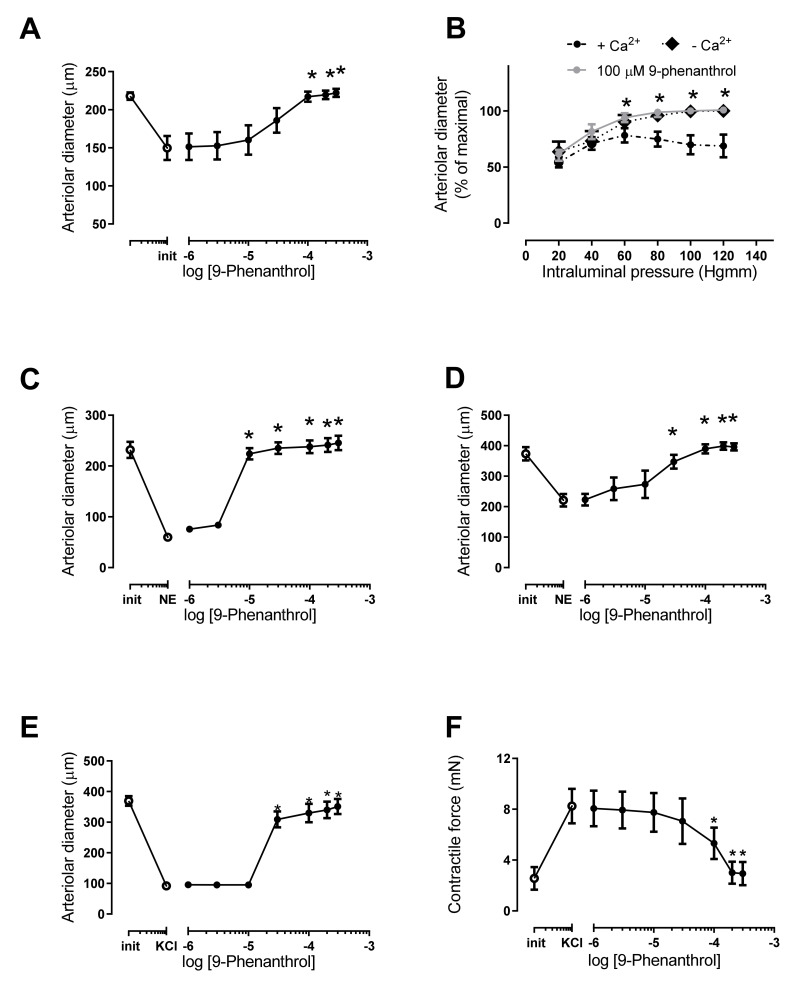
TRPM4 inhibitor 9-phenanthrol completely antagonized myogenic tone and both norepinephrine and KCl evoked vasoconstrictions. Pressure myography of rat skeletal muscle (**A**–**C**) and mesenteric (**D**,**E**) arteries was performed. Basilar arteries (**F**) were tested on an isometric force measurement system. (**A**) Myogenic tone was allowed to develop at 80 mmHg intraluminal pressure (first symbol: before, second symbol: after myogenic tone development), then 9-phenanthrol was added to the organ bath at the indicated concentrations. (**B**) Myogenic tone was first assessed in the presence of Ca^2+^ on skeletal muscle arterioles by increasing intraluminal pressure in 20 mmHg increments from 20 mmHg to 120 mmHg. Vessels were then incubated with 100 µM 9-phenanthrol and the same procedure was repeated. Vessels were then thoroughly washed, and diameter was also recorded in the absence of Ca^2+^. (**C**) Skeletal muscle arterioles were preconstricted with 10 µM norepinephrine (first symbol: before, second symbol: after norepinephrine, NE); then, 9-phenanthrol was added to the organ bath at the indicated concentrations. (**D**) Mesenteric arteries were preconstricted with 10 µM norepinephrine (first symbol: before, second symbol: after norepinephrine, NE); then, 9-phenanthrol was added to the organ bath at the indicated concentrations. (**E**) Mesenteric arteries were preconstricted with 80 mM KCl; then, 9-phenanthrol was added to the organ bath. (**F**) Basilar arteries were preconstricted with 80 mM KCl; then, 9-phenanthrol was added to the organ bath. Graphs show the mean ± SEM. Significant differences (*p* < 0.05) from the initial diameter (panels **A**,**C**,**D**) or from the diameter in the presence of Ca^2+^ (Panel **B**) are labeled by the asterisks (*). Five (*n* = 5) animals were used for each panels (**A**–**E**), while four animals were used for panel (**F**).

The skeletal muscle arteries showed a dilation upon 9-phenanthrol treatment (from 150 ± 16 µm to 217 ± 7 µm, *n* = 5, *p* = 0.02) at 100 µM concentration (Figure 2A). This represented the loss of spontaneous myogenic tone (which was 31% of the passive diameter). The pressurized skeletal muscle arteries developed a myogenic response to elevations in intraluminal pressure (note the lower diameters in the presence of Ca^2+^ in Figure 2B). This myogenic response was missing in the presence of 100 µM 9-phenanthrol (Figure 2B).

### 2.3. TRPM4 Inhibition Completely Antagonized Norepinephrine Evoked Vasoconstriction

The effect of TRPM4 inhibition was tested on norepinephrine-evoked vasoconstriction of various arteries. Norepinephrine-evoked vasoconstriction (vessel diameter decreased from 232 ± 16 to 60 ± 2 µm) was completely antagonized by 9-phenanthrol from a concentration of 10 µM (diameter returned to 238 ± 13 µm, Figure 2C) in skeletal muscle arteries. A similar response was observed in mesenteric arteries (norepinephrine constriction from 373 ± 22 to 221 ± 21 µm), with complete antagonism (diameter returned to 390 ± 15 µm) by TRPM4 inhibition (Figure 2D).

### 2.4. TRPM4 Inhibition Completely Antagonized Vasoconstriction Caused by High Extracellular KCl Concentration-Evoked Depolarization of the Smooth Muscle Cells

The mesenteric arteries were preconstricted with KCl, and 9-phenanthrol-evoked vasodilation was measured by pressure myography. The diameters of the arteries decreased from 369 ± 16 µm to 92 ± 10 µm (*p* < 0.05) upon KCl constriction. This constriction was completely antagonized by 9-phenanthrol (the diameters returned to 309 ± 26 µm at 30 µM, *n* = 5, Figure 2E). A similar effect was noted on isolated basilar arteries measured by isometric force measurements on arteriolar rings. The tension of the arteries increased from 2.6 ± 0.9 mN to 8.3 ± 1.4 mN upon KCl stimulation. This constriction was completely antagonized by 9-phenanthrol as the contractile force returned to 2.9 ± 0.9 mN at the highest-applied 9-phenanthrol concentration (300 µM, *n* = 4, Figure 2F).

### 2.5. TRPM4 Inhibition Completely Antagonized Norepinephrine-Evoked Vasoconstriction in the Absence of Endothelium

**Figure 3:**
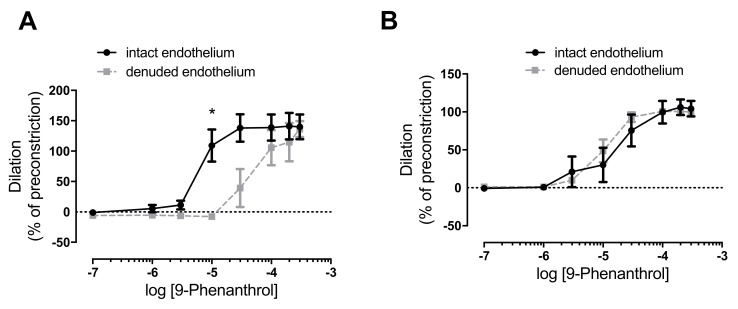
TRPM4 inhibitor 9-phenanthrol completely antagonizes norepinephrine (Panel **A**) and KCl evoked vasoconstrictions (Panel **B**), irrespectively of the endothelium. Pressure myography of rat skeletal muscle (**A**) and mesenteric arteries (**B**) was performed. Endothelium was removed via intraluminal perfusion of air and distilled water (denuded endothelium). Vessels were preconstricted with 10 µM norepinephrine; then, 9-phenanthrol was added in the indicated concentrations. Significant difference is indicated by the asterisk (*, *p* < 0.05, Sidak’s multiple comparison) vs. dilation in arteries with intact endothelium. Mean ± SEM are plotted on the graphs. Five (*n* = 5) rats were used for the experiments.

A role of the endothelium was tested in vasodilation upon TRPM4 inhibition. On a subset of vessels, the endothelium was removed to assess the endothelial contribution to vasodilation caused by 9-phenanthrol. Endothelium denudation was confirmed by blunted acetylcholine dilation (data not shown). The arteries were then treated with norepinephrine and then with 9-phenanthrol, as before (such as in Figure 2C,D). Endothelium denudation shifted the 9-phenanthrol concentration curve to the right in the skeletal muscle arteries (more 9-phenanthrol was needed to antagonize norepinephrine constriction, Figure 3A), while it was without effects in mesenteric arteries (Figure 3B).

### 2.6. TRPM4 Inhibition Antagonized Norepinephrine-Evoked Vasoconstriction on Skeletal Muscle Arteries by Decreasing Intracellular Ca^2+^ Concentration in Vascular Smooth Muscle Cells

**Figure 4:**
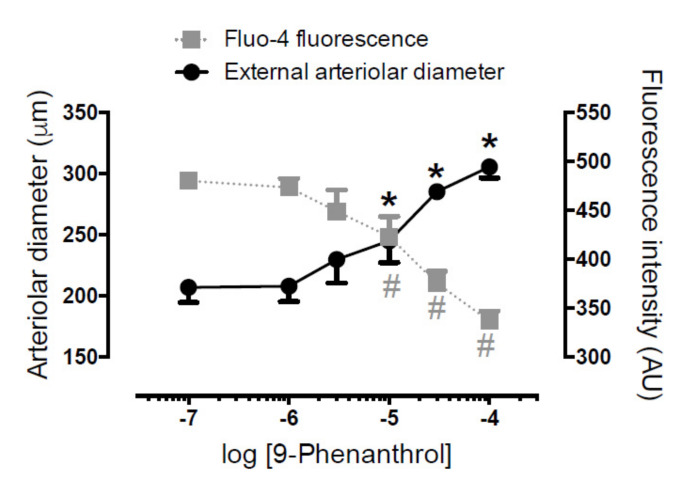
TRPM4 inhibitor 9-phenanthrol completely antagonizes norepinephrine-evoked increases in intracellular Ca^2+^ concentrations and vasoconstrictions in skeletal muscle arteries. Pressure myography was performed on rat skeletal muscle arterioles. Blood vessels were loaded with Ca^2+^-sensitive fluorescent dye (fluo-4AM). Arterioles were pretreated with 10 µM norepinephrine, which led to vasoconstriction, together with an increase in fluo-4 fluorescence of the vessel. 9-Phenanthrol was added to the organ bath, while external arteriolar diameter and wall fluorescence were measured and plotted on the graph. Black asterisks (*): *p* < 0.05 (Dunnett’s multiple comparison) vs. initial diameter. Grey hashes (^#^): *p* < 0.05 (Dunnett’s multiple comparison) vs. initial fluorescence. Symbols and bars are Mean ± SEM. Six (*n* = 6) animals were used.

On a subset of skeletal muscle arterioles, changes of intracellular Ca^2+^ concentration (measured in the arteriolar wall) were measured in parallel with the arteriolar diameter. Arterioles were preconstricted with norepinephrine, which led to a decrease in arteriolar diameter (from 296 ± 16 µm to 206 ± 13 µm, *n* = 6, *p* < 0.05) and an increase in fluorescence (from 237 ± 71 AU to 480 ± 121 AU, *n* = 6, *p* < 0.05). These changes in diameter and fluorescence were completely antagonized by 9-phenanthrol (diameter returned to 305 ± 9 µm, fluorescence intensity to 337 ± 46 AU, *n* = 6, Figure 4).

### 2.7. Immunohistochemistry Confirmed Expression of TRPM4 in Arteries Used in the Functional Assays

**Figure 5:**
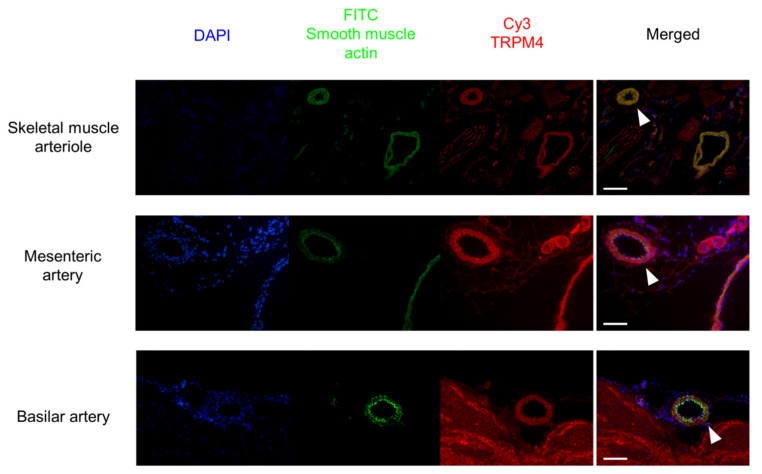
TRPM4 is expressed in the smooth muscle layers of blood vessels used in the functional studies. Tissue sections were prepared from skeletal muscle, mesentery, and brainstem. DAPI: nuclei (blue); FITC smooth-muscle actin: smooth muscle cells (green); Cy3-TRPM4 labeling (red). White arrowheads point to the blood vessels used in the functional studies on merged (overlayed) images. Scale bar = 100 µm. Histological sections were prepared from three animals. It is not known whether the figures for separate arterial beds represent the same or different animals.

Tissue sections were prepared from *m. gracilis* (containing the superficial blood vessels), mesentery (containing the second-order branches of the mesenteric artery), and brainstem (containing the superficial basilar artery). All blood vessels showed TRPM4 staining, which was colocalized with the smooth-muscle actin staining (Figure 5). Note that brain tissue also showed diffuse staining for TRPM4, while TRPM4 expression was more specific for smooth muscle compared to other tissues.

### 2.8. The Ca^2+^ Ionophore A23187 Acts as a TRPM4 Agonist Which Is Competitively Antagonized by 9-Phenanthrol in Skeletal Muscle Arteries

**Figure 6:**
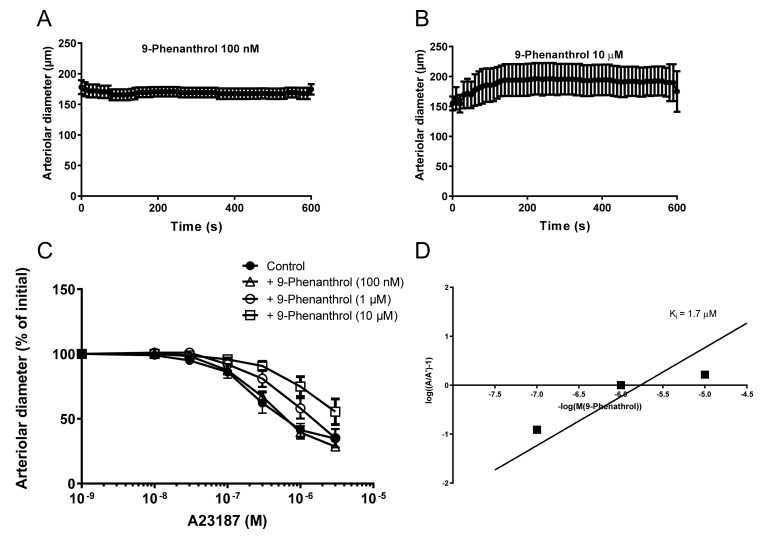
The Ca^2+^ ionophore A23187 is a competitive agonist of TRPM4. Skeletal muscle (*m. gracilis*) arteries were isolated from the rat and the changes in vascular diameter were recorded upon 9-phenanthrol addition into the organ bath (Panel **A**,**B**, *n* = 5). Note, 9-phenanthrol had no effects at a concentration of 100 nM (maintained myogenic tone and vascular diameter, panel **A**), while it resulted in a loss of myogenic tone (and in an increased diameter) at higher (shown at 10 µM) concentrations (Panel **B**). In individual arteries, the effects of 9-phenanthrol (applied concentrations are labeled) on A23187-evoked vasoconstrictions were also tested (Panel **C**). The apparent EC50 values were plotted on a Schild plot (Panel **D**) to yield the stability constant of 9-phenanthrol to bind to the A23187 binding sites in vascular preparations. Note, these results implicated a common receptor for 9-phenanthrol and A23187 (e.g., TRPM4) in this system. Twenty (*n* = 20) animals were used for these studies.

The skeletal muscle arteries were treated with 9-phenanthrol to test the duration of the effects. 9-Phenanthrol evoked a long-lasting concentration-dependent vasodilation (Figure 6A,B). The Ca^2+^ ionophore A23187 was also tested for vascular responses. The A23187 evoked a concentration-dependent vasoconstriction in the skeletal muscle arteries (Figure 6C). This A23187-evoked vasoconstriction was competitively inhibited by 9-phenanthrol, applied at 0.1–10 µM concentration. The shift in apparent EC_50_ values for A23187 in the presence of different 9-phenanthrol concentrations (Schild plot, Figure 6D) suggested a K_i_ value of 1.7 µM (pA2 value was −5.77) for 9-phenanthrol to bind to the A23187 receptor.

## 3. Discussion

The current study was designed to investigate the contribution of TRPM4 to vascular constriction in various isolated arteries and in the systemic circulation. TRPM4 inhibition with 9-phenanthrol caused a significant, transient decrease in both systolic and diastolic blood pressure, in addition to a concurrent drop in the rat’s heart rate. The latter result accords with other studies, where TRPM4 expression was reported in the myocardium [21], especially in nodal and conductive tissue [22,23,24]. The decrease in blood pressure can be explained by the decreased heart rate alone (confirmed here), although a contribution from blunted vascular responses cannot be ruled out.

9-Phenanthrol was reported to play an important role in the myogenic response of intracranial arteries [4]. Here, we confirmed this finding and extended it to peripheral arteries. Skeletal muscle arteries having a (high) myogenic response similar to that of intracranial arteries also dilated to their maximal diameter in the presence of 9-phenanthrol. Moreover, intraluminal pressure elevations did not result in vasoconstrictions, suggesting that myogenic tone in skeletal muscle arteries was completely antagonized by TRPM4 inhibition, similar to that reported for intracranial arteries [4].

TRPM4 inhibition by 9-phenanthrol also completely antagonized the vasoconstriction caused by norepinephrine in both skeletal muscle and mesenteric arteries in this study. This 9-phenanthrol-mediated dilation of the constricted arteries was not affected by endothelium denudation (although higher 9-phenanthrol concentrations were required in skeletal muscle arteries). The effect of TRPM4 inhibition by 9-phenanthrol was also tested on mesenteric and basilar arteries, in which vasoconstriction can be evoked by direct depolarization of the smooth muscle cell membrane with KCl. The TRPM4 inhibitor 9-phenanthrol significantly dilated KCl-preconstricted vessels, similar to the dilation in agonist-preconstricted arteries. Antagonism of depolarization-evoked vasoconstriction suggested that TRPM4 may play a role in the regulation of L-type Ca^2+^ channels.

The effects of TRPM4 inhibition by 9-phenanthrol were tested on smooth muscle cell Ca^2+^ homeostasis in skeletal muscle arterioles. Vessels were loaded with a Ca^2+^-sensitive fluorescent dye (Fluo-4 AM) and were treated with norepinephrine to evoke vasoconstriction (resulting in an increase in intracellular Ca^2+^ concentration). Administration of 9-phenanthrol completely antagonized the vasoconstriction evoked by norepinephrine, by reversing the increases in intracellular Ca^2+^ concentrations. These results suggest that 9-phenanthrol is acting on the regulation of intracellular Ca^2+^ concentration. This is in accordance with the observed TRPM4 expression in the smooth muscle cells of blood vessels.

The results of this study confirm some of the previous studies. In particular, 9-phenanthrol caused vasodilation in cerebral arteries with myogenic or KCl-evoked vasoconstrictions [4,18]. Further, downregulation of TRPM4 also caused deterioration of purinergic receptor agonist-evoked vasoconstriction in cerebral parenchymal arteries [5]. This study adds to these findings, reporting that these effects could be observed in peripheral arteries upon the pharmacological inhibition of TRPM4. The results obtained from measurements on nonmyogenic vessels are similar to the results presented by Garland et al. [16], where the pretreatment of mesenteric artery rings with 9-phenanthrol inhibited norepinephrine-evoked vasoconstriction, which was not observed in the absence of the endothelium. Hence, the study considered the 9-phenanthrol-mediatied vasodilation mostly a result of the intermediate conductance of Ca^2+^-activated K^+^ channel (K_Ca3.1_) activation on the endothelium and consequential hyperpolarization of the smooth muscle cells through the myoendothelial junctions [16]. We showed in this study that this mechanism cannot be generalized. It needs to be mentioned that Burris et al. [25] challenged the specificity of smooth muscle cell TRPM4 inhibition with 9-phenanthrol. In particular, 9-phenanthrol inhibited the myogenic tone in cerebral vessels by concurrent inhibition of TRPM4 and TMEM16A channels. TMEM16A is a chloride channel that is known to contribute to the myogenic response of cerebral arteries [26].

Our results suggest that TRPM4 inhibitor 9-phenanthrol can indirectly decrease intracellular Ca^2+^ concentration, interfering with the development of myogenic tone or agonist-evoked vasoconstriction. In line with this, we used the Ca^2+^ ionophore A23187 to pinpoint the site of action of 9-phenanthrol. The A23187-mediated vasoconstriction was antagonized by 9-phenanthrol. Importantly, the antagonism was competitive. This suggests that A23187 acted on the 9-phenanthrol receptor, suggesting a direct targeting of TRPM4. As a matter of fact, A23187 was identified as a TRPM4 agonist here, with an apparent EC_50_ value of about 100 nM. It is important to note that this A23187-mediated vasoconstriction was competitively inhibited by 9-phenanthrol, yielding a Ki value of 1.7 µM for 9-phenanthrol. This suggests that A23187 may directly bind to the 9-phenanthrol binding site in TRPM4, hence being the first potent specific agonist of TRPM4.

TRPM4 acted as a key Ca^2+^-signal amplifier, since 9-phenanthrol completely inhibited all measurable increases in the intracellular Ca^2+^ concentration in the vessel wall. This suggests that TRPM4 provides a positive feedback mechanism for intracellular Ca^2+^ elevations in the arteriolar wall. Another important novel finding was that the low concentration of the Ca^2+^ ionophore A23187 seems to act as a specific activator of smooth muscle TRPM4. These results suggest that TRPM4 represents a therapeutic target for cardiovascular diseases such as hypertension. In relation to this, the identification of A23187 as a nanomolar-affinity direct agonist of TRPM4 provides a lead structure for molecular pharmacology of TRPM4.

The current study has limitations. We did not perform an in-depth electrophysiological characterization of the ion channels involved in vascular constrictions. 9-Phenanthrol may also act on targets other than TRPM4, as relatively high concentrations were needed in the vascular studies. Moreover, the studies were performed on rats having a wide age range (from 9 to 15 weeks), which may have affected their TRPM4 mediated responses.

## 4. Materials and Methods

### 4.1. Animal Housing and Experimental Protocol Approval

Male Wistar (Crl:WI) rats were acquired for this study from Toxi-Coop (Budapest, Hungary). The experimental protocol was approved by the Animal Welfare Committee of the University of Debrecen (DEMÁB/63-1/2013).

### 4.2. Invasive Measurement of Blood Pressure

Rats (250–450 g) were anesthetized with an intraperitoneal injection of thiopental (Braun, 100 mg/bwkg). Total anesthesia was confirmed by the absence of any reactions to firm pressure at the base of the rats’ tails. This lack of responsiveness was tested throughout the experiments. If any responses were observed, an additional maintenance dose (20 mg/bwkg) of thiopental was administered intraperitoneally. After total anesthesia was achieved, the trachea, jugular vein, and carotid artery were cannulated with polyethylene tubing. Blood pressure was measured by inserting a cannula into the left carotid artery. 9-Phenanthrol was administered into the jugular vein cannula. Consecutive doses of 9-phenanthrol were administered when the blood pressure and heart rate stabilized from the previous treatment, usually after 1–3 min. An ECG was recorded from the limbs; the temperature was taken in the rectum. All parameters were recorded using a rodent surgical monitoring system (Experimetria ELS-01, Experimetria Ltd., Budapest, Hungary). The animals were euthanized after the experiments. Different rats were used for the in vivo and in vitro studies.

### 4.3. Pressure Myography of Arteries

The rats were anesthetized as described above. After total anesthesia was achieved, the animals were exsanguinated via a transverse cut on the thoracic aorta. Skeletal muscle, mesenteric arcade, and whole-brain samples were collected and quickly transferred to ice-cold, oxygenated, Ca^2+^-free Krebs–Henseleit buffer containing (mM): 110 NaCl, 5 KCl, 1 MgSO_4_, 1 KH_2_PO_4_, 5 glucose, and 24 NaHCO_3_, pH = 7.4.

The proximal caudal femoral artery (passive diameter: 200–250 µm) was dissected from the skeletal muscle (*m. gracilis*). From the mesenteric arcades, the 2nd-order-segment mesenteric artery was collected (passive diameter: 300–400 µm). From the whole-brain samples, a segment of the basilar artery was removed (the diameter when stretched by the wires: 1.5–2 mm).

The skeletal muscle and mesenteric vessels were transferred to the organ bath of a pressure myograph (CH-1, Living Systems Instrumentation, St. Albans City, VT, USA). After cannulating the vessels, the buffer was replaced with Ca^2+^-containing (2.5 mM) Krebs–Henseleit buffer. The cannulated vessels were then allowed to equilibrate for 60 min at 37 °C under 80 mmHg pressure with a constant gas flow (85% N_2_, 10% O_2_, 5% CO_2_) directed into the buffer. Smooth muscle function was tested with norepinephrine (Sigma-Aldrich, St. Louis, MO, USA, 1 nM to 10 µM) on both mesenteric and skeletal muscle arteries. On the skeletal muscle vessels, the endothelial function was tested with acetylcholine (Sigma-Aldrich, St. Louis, MO, USA, 1 nM to 10 µM) after the development of myogenic tone. Endothelium-mediated dilation was measured after the myogenic tone had developed in the skeletal muscle arterioles, and was measured following preconstriction with norepinephrine (10 µM) in the mesenteric arteries. Vessels that had intact smooth muscle and endothelial responses were used for further experiments. The effect of 9-phenanthrol (Sigma-Aldrich, 100 nM to 300 µM) was measured after the development of myogenic tone (skeletal muscle arteries), or after preconstriction with 10 µM norepinephrine (skeletal muscle arteries and mesenteric arteries) or 80 mM KCl (mesenteric arteries).

On a subset of the skeletal muscle arteries, to further investigate the effect of TRPM4 inhibition on myogenic tone, the vessels were incubated with 100 µM 9-phenanthrol at 20 mmHg intraluminal pressure. The pressure was then raised in 20 mmHg increments up to 120 mmHg, and the vessel diameter was recorded 4 min after the pressure had been raised. After thorough washing with Ca^2+^-free Krebs–Henseleit buffer, the same procedure (in the same buffer without 9-phenanthrol) was repeated.

The endothelium was denuded by perfusing the vessel with air (30–60 s) followed by distilled water (60–120 s) in a subset of skeletal muscle and mesenteric arteries. The dilative effect of acetylcholine was measured before and after this process, and only those vessels that showed no dilation upon acetylcholine after the procedure were used for further experiments. All endothelium-denuded arteries exhibited at least 80% constriction when tested with 10 µM norepinephrine (compared to the response before denudation).

Measurement of the Ca^2+^ concentration within the vessel wall was also performed. These vessels were incubated for 90 min in Ca^2+^-containing Krebs–Henseleit solution supplemented with 4.5 µM fluo-4-AM (Thermo Fisher Scientific, Waltham, MA, USA), 100 µM probenecid, 0.5% bovine serum albumin, and 1× PowerLoad (Thermo Fisher Scientific, Waltham, MA, USA). After washing the vessels with Krebs–Henseleit buffer, the arteries were illuminated at 470 nm and the fluorescence signals were detected at 510 nm by video microscopy (InCyt Im2 system, Intracellular Imaging Inc., Cincinnati, OH, USA). The intracellular Ca^2+^ concentration (fluorescence intensity in the vessel wall) and the external arteriolar diameter were determined using the fluorescent images.

### 4.4. Wire Myography of Arteries

The basilar artery segments (about 4 mm in length) were transferred to the organ chamber of a wire myograph (DMT-510A, Danish Myo Technologies, Hinnerup, Denmark). After the artery rings were secured to myograph clamps, the buffer was replaced with Ca^2+^-containing Krebs–Henseleit buffer with a constant carbogen gas (95% O_2_, 5% CO_2_) flow directed into the bath. A pre-stretching protocol was performed to set the vessel rings to their physiological tension [27]. The vessel rings were then allowed to equilibrate for 60 min. The vessels’ smooth muscle viability was tested with serotonin (5-hydroxy-tryptamine, Sigma-Aldrich, 1 nM to 10 µM) and KCl (10 to 80 mM). The effect of 9-phenanthrol (Sigma-Aldrich, 100 nM to 300 µM) was measured after preconstriction with 80 mM KCl.

### 4.5. Immunohistochemistry

Tissue samples were taken from the male Wistar rats from the areas mentioned above. Samples were embedded in CryoMatrix resin (Thermo Fisher Scientific, Waltham, MA, USA) and were frozen over the surface of boiling liquid nitrogen. Frozen sections (10 µm) were allowed to air dry (4–5 min), were then fixated in acetone (5–10 min at 4 °C), and air dried. The sections were then stored at 4 °C until the immunohistochemistry was performed. The Sections were washed with PBS (without Ca^2+^/Mg^2+^ from Invitrogen), incubated in methanol for 20 min, washed again in PBS (5 min), and blocked in goat serum (150 µL normal goat serum (Dako, Glostrup, Denmark) in 10 mL PBS) for 20 min. The sections were then incubated with rabbit anti-TRPM4 (Abcam ab104572) and mouse antismooth-muscle actin (Novocastra NCL-Sma, Leica Biosystems, Buffalo Grove, IL, USA) at dilutions of 1:50, followed by washing in PBS. Then, the tissue sections were incubated with the secondary antibodies (goat anti-rabbit-biotin and goat anti-mouse-biotin, both 1:200, from Jackson Immuno Research Labs, West Grove, PA, USA) for 30 min. The specimens were washed with PBS, and biotin was reacted with Cy3-straptavidin (Jackson Immuno Research Labs, West Grove, PA, USA). Finally, the sections were washed and covered in a DAPI-containing medium (Vector Laboratories, Burlingame, CA, USA) before placing the coverslips. Fluorescent pictures were taken with a fluorescent microscope (NIKON, Minato, Japan).

### 4.6. Statistical Analysis

Statistical analysis was performed with GraphPad Prism 8.4.3. The Kolmogorov–Smirnov test was used to determine the normal distribution of the data. The means ± SEM are reported. On normally distributed data, one- or two-way repeated measures ANOVA were performed with a corresponding post hoc test (Dunnett’s, Tukey’s, or Sidak’s) to compare values measured after treatments against the values measured at the beginning of the experiment or against the other groups. A nonparametric Friedman test was performed with Dunn’s post-test on non-normally distributed data. Results were considered as statistically significant at *p* < 0.05.

## Data Availability

The data analyzed and presented in this study are available from the corresponding author on request.
